# Preparing n-of-1 Antisense Oligonucleotide Treatments for Rare Neurological Diseases in Europe: Genetic, Regulatory, and Ethical Perspectives

**DOI:** 10.1089/nat.2021.0039

**Published:** 2022-04-19

**Authors:** Matthis Synofzik, Willeke M.C van Roon-Mom, Georg Marckmann, Hermine A. van Duyvenvoorde, Holm Graessner, Rebecca Schüle, Annemieke Aartsma-Rus

**Affiliations:** ^1^ Department of Neurodegenerative Diseases, Hertie-Institute for Clinical Brain Research and Center of Neurology, University of Tübingen, Tübingen, Germany.; ^2^ Center for Neurodegenerative Diseases (DZNE), Tübingen, Germany.; ^3^ Department of Human Genetics, Leiden University Medical Center, Leiden, The Netherlands.; ^4^ Institute of Ethics, History and Theory of Medicine, Ludwig Maximilians University Munich, Munich, Germany.; ^5^ Department of Clinical Genetics, Leiden University Medical Center, Leiden, The Netherlands.; ^6^ Institute of Medical Genetics and Applied Genomics, University of Tübingen, Tübingen, Germany.; ^7^ Center for Rare Diseases, Tübingen, Germany.

**Keywords:** antisense oligonucleotides, n-of-1, rare diseases, rare neurological diseases, regulatory, policy, ethics

## Abstract

Antisense oligonucleotide (ASO) therapies present a promising disease-modifying treatment approach for rare neurological diseases (RNDs). However, the current focus is on “more common” RNDs, leaving a large share of RND patients still without prospect of disease-modifying treatments. In response to this gap, n-of-1 ASO treatment approaches are targeting ultrarare or even private variants. While highly attractive, this emerging, academia-driven field of ultimately individualized precision medicine is in need of systematic guidance and standards, which will allow global scaling of this approach. We provide here genetic, regulatory, and ethical perspectives for preparing n-of-1 ASO treatments and research programs, with a specific focus on the European context. By example of splice modulating ASOs, we outline genetic criteria for variant prioritization, chart the regulatory field of n-of-1 ASO treatment development in Europe, and propose an ethically informed classification for n-of-1 ASO treatment strategies and level of outcome assessments. To accommodate the ethical requirements of both individual patient benefit and knowledge gain, we propose a stronger integration of patient care and clinical research when developing novel n-of-1 ASO treatments: each single trial of therapy should inherently be driven to generate generalizable knowledge, be registered in a ASO treatment registry, and include assessment of generic outcomes, which allow aggregated analysis across n-of-1 trials of therapy.

## Introduction

Rare diseases are defined as diseases that occur with an incidence of 1 in ≤2,000 in Europe. While each of these diseases is individually rare, there are an estimated 6,000–8,000 rare diseases and ∼80% of those have a genetic cause [1]. It is estimated that 6%–8% of the European population are affected by a rare disease [2]. While some rare diseases are comparatively common with an incidence ranging between 1:2,000 and 1:10,000, the majority of rare diseases are “ultrarare” with incidences of <1 in 100,000 individuals [3]. For some of the more common rare diseases—such as Duchenne muscular dystrophy with an incidence of 1 in 5,000 newborn boys—variant specific therapies have been developed, which apply to subsets of patients only. The stop codon read through compound approved by European Medicines Agency (EMA) applies to ∼10% of variants carrying a nonsense variant, while antisense oligonucleotide (ASO)-mediated exon skipping compounds approved by the Food and Drug Administration (FDA) are applicable to 8%–14% of patients [4]. This variant-specific stratification results in the fact that even some of the treatment approaches for comparatively common diseases ultimately target small “ultrarare” cohorts.

For patients requiring individualized treatment due to the rarity of their disease or variant, there is little or no commercial interest for developing treatments, resulting in a high unmet need for this group of patients. At the same time, for some of these patients it can be anticipated that individualized treatment leads to an improvement in quality of life. This was highlighted by the development of Milasen in 2018 in the United States, where a team from Boston developed an ASO individualized for a single patient—6-year-old Mila Makovec—with neuronal ceroid lipofuscinosis 7 (CLN7, a form of Batten's disease) caused by a unique cryptic splicing variant in the *SLN7* gene [5]. Since then, n-of-1 ASOs have been developed for several other individual patients through accelerated regulatory pathways, including an individualized ASO for a specific ataxia-telangiectasia (AT) mutation (“Atipeksen”) and one for a specific Amyotrophic Lateral Sclerosis (ALS) causing mutation in *FUS* (“Jacifusen,” named after 26-year-old Jaci Hermstad, diagnosed with *FUS*-associated ALS in February 2019 and first patient treated with this ASO, which was already under development by Ionis Pharmaceuticals). In this perspective paper, we will focus on highly individualized splice modulating therapies for rare neurological diseases (RNDs) for even single subjects with possibly private variants (n-of-1 treatment), and will outline scientific, clinical, legal, regulatory, and ethical considerations for preparing the path toward such n-of-1 treatments from a European perspective.

## Genetic Perspectives: What Can Guide a Promising n-of-1 Approach in RNDs?

### ASO-mediated splicing modulation

ASOs are attractive tools for the treatment of RNDs—which are often multisystemic and affect widespread central nervous system (CNS)—as they allow high exposure across the CNS after intrathecal injections and good uptake by cells in the CNS after infrequent maintenance dosing (eg, every 4 months) [6].

Splice modulating ASOs have a proven track record for treatment of neurological diseases. Nusinersen, an ASO approved by both FDA and EMA, increases inclusion of exon 7 in SMN2 transcript, thus resulting in increases in SMN proteins, which have therapeutic effects for all types of spinal muscular atrophy [7].

**FIG. 1.**
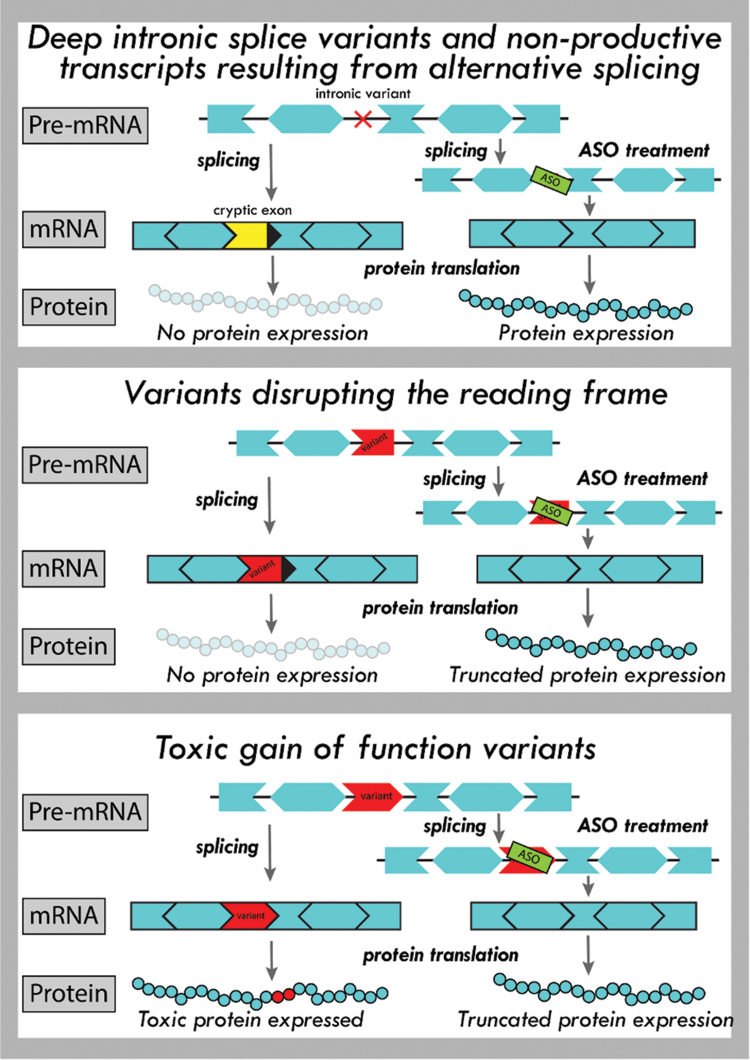
Mechanisms of genetic variant types and their targeting by splice modulation ASOs (examples). *Top panel*: deep intronic pathogenic variants can result into part of an intron being aberrantly included into the mRNA transcripts, thus abolishing protein function. ASOs can prevent inclusion of these cryptic exons. Similarly, for some genes, alternative splicing occurs where an exon is included, which leads to nonproductive transcripts. ASOs can target these nonproductive splicing events, preventing inclusion of these alternative exons and thus increasing productive mRNA and protein (TANGO approach). *Middle panel*: pathogenic variants can disrupt the open reading frame. ASO-mediated exon skipping can restore the reading frame, allowing production of an internally deleted, partially functional protein. *Bottom panel*: pathogenic variants can result in the production of a toxic protein. Skipping the exon harboring the variant will result in the production of a shorter, nontoxic protein. ASO, antisense oligonucleotide. Color images are available online.

There are several ways ASOs can modulate splicing (Fig. 1 and Table 1). As mentioned above, they can induce exon inclusion or exon skipping to restore protein production [8,9]. Other possibilities are removal of toxic-gain-of-function variants from genes [10,11], increasing the endogenous expression of productive mRNAs over naturally occurring nonproductive mRNAs [12], switching from detrimental to protective protein isoforms [13], or the correction of aberrant intron inclusion due to deep intronic variants [5,14].

**Table 1.** Overview of Different Methods Splice Modulating Antisense Oligonucleotides Can Be Used for Neurological Disorders

Method	Example	Status
Exon inclusion to restore protein production	Nusinersen for spinal muscular atrophy	EMA and FDA approved
Exon skipping to induce production of partially functional protein	Eteplirsen, golodirsen, viltolarsen, and casimersen for Duchenne muscular dystrophy	FDA approved
Exon skipping to increase amount of productive transcript	STK-001 for Dravet syndrome	Phase 1 clinical trial (NCT04442295)
Exon skipping to reduce amount of toxic transcript or protein	Skipping toxic repeat containing exon 10 in ataxin-3 for spinocerebellar ataxia type 3 (SCA3)	Preclinical
Modulating alternative splicing from detrimental to protective isoform	Converting detrimental three repeat (3R) tau to beneficial 4R tau in frontotemporal dementia	Preclinical
Correction of cryptic splicing variants	Steric block to prevent the use of a cryptic splice site, for example, due to a deep intronic variant (milasen for cryptic splicing variant of CLN7 gene or deep intronic variant in BPAN or OPA1) or creation of a novel splice-donor (as in AT, atipeksen) or splice-acceptor site	IND given for milasen and atipeksen by FDA for individualized treatment; all other examples: preclinical

Designing and optimizing splice modulating ASOs so far is a matter of trial and error. Industry generally designs many consecutive ASOs to cover an entire exon (exon walk). This is often not feasible in an academic setting, where a more targeted approach is used, here leveraging guidelines that have been designed based on retrospective analysis of effective and ineffective exon skipping ASOs [15]. While these guidelines help identify likely target sequences and a software tool to facilitate exon skipping ASO design has been produced [16], this is not yet an exact science and sometimes two to three rounds of optimization are needed to identify a sufficiently effective ASO *in vitro*.

### Prioritization criteria for variants that can be targeted by splice modulating ASOs

Not all unique pathogenic variants can be targeted by splice modulating ASOs. The deciding question is whether ASO treatment can restore production of functional protein, and this has to be determined for each variant (Table 2).

**Table 2.** Inclusion Criteria for Splice Modulation Antisense Oligonucleotide Therapy Development: Antisense Oligonucleotide Strategy/Mutation Mechanism

	DNA variant prioritization (most optimal from left to right)				
**Effect of DNA variant**	Activation of **intronic** cryptic acceptor/donor splice site	Activation of **exonic** cryptic acceptor/donor splice site	**Exonic** frameshift or nonsense variants	**Exonic** variant causing toxic gain of protein function	**Exonic** missense variant causing loss of protein function
**ASO mode of action**	Prevent cryptic exon inclusion	Restore normal transcript	Exon exclusion to restore ORF	Exclusion of the exon containing the variant	Exclusion of the exon containing the variant
**Essential criteria**	Target >50 bp from exon/intron boundary	Target >10 bp from canonical splice site	Excluded exon(s) do not disturb the ORF	Excluded exon(s) do not disturb the ORF	Excluded exon(s) do not disturb the ORF
**Result on protein level**	Restore normal protein expression	Restore normal protein expression	Production of (internally) truncated protein that retains most important wild-type function	Production of (internally) truncated protein that retains most important wild type	Production of (internally) truncated protein that retains most important wild type

Deep intronic pathogenic variants are excellent targets for ASO-mediated splice modulation, as skipping the cryptic exon will restore the normal transcript and normal protein production. However, intronic variants can also affect the canonical donor or acceptor splice sites. These variants often result in skipping of one or more exons. If this disrupts the reading frame, ASO can be used to induce a flanking exon(s) to restore the reading frame. It is then imperative that the protein produced is still functional lacking the domain(s) encoded by the missing exons. For most proteins, this will not be the case. However, there are proteins with partially redundant domains such as dystrophin and collagens, where internally deleted proteins are partially functional [17,18].

For variants located within exons, different questions have to be asked based on the type of variant. For variants that disrupt the reading frame, cause a premature nonsense codon, or result in toxic gain of function, skipping the mutated exon will bypass the variant. For in-frame exons, this will allow the production of an internally deleted protein. Here the question again will be whether that protein is functional or not. If the exon is out of frame one would have to skip multiple exons to generate an in-frame transcript. Skipping multiple exons is much more challenging from a drug development perspective as it requires a combination of ≥2 ASOs [19]. Exonic variants that disrupt splicing have to be evaluated on a case-by-case basis. For those that involve the generation of an intraexonic splice site, causing a deletion of part of the exon from the transcript, ASOs may restore normal splicing by enhancing use of the standard splice site. However, it is uncertain whether the binding of the ASO to the transcript will interfere with protein translation. For those that result in exon skipping, the same considerations apply as for variant directly involving the donor or acceptor splice sites.

Missense variants also have to be evaluated on a case-by-case basis. However, they will mostly not be eligible for ASO-mediated splicing modulation. This is because the mere fact that a missense variant is pathogenic implies that this variant is in a crucial domain of the protein. Therefore, skipping the exon carrying the variant will result in protein lacking that domain, which most likely will not be functional—unless the protein is tolerant to (ASO-induced) loss of function of that very particular protein domain.

For diseases caused by haploinsufficiency, targeted augmentation of nuclear gene output (TANGO) may be an option [12] (Fig. 1). This approach relies on the presence of nonproductive alternatively spliced transcripts, which contain an out-of-frame exon or an exon with a stop codon. Skipping such an exon will reduce the amount of nonproductive splicing events, while increasing the amount of productive transcript and consequently the amount of protein. This approach does, however, critically depend on whether nonproductive transcripts are produced in brain or eye. This is the case for many genes involved in RNDs [12].

Notably, these are ways splice modulating ASOs can be used *in principle*. Nevertheless, most of these approaches have not yet been tested in clinical trials; and even for those that have been shown to work for one gene transcript and one disease, there is no guarantee that similar ASO splice modulations will work when targeting another transcript. This is why in each case one has to not only establish if inclusion of the targeted exonic region is indeed prevented, but also whether the resulting protein is functional and/or less toxic in preclinical models.

### Finding cryptic splicing variants

Those cryptic splice site variants, where intronic variants induce the inclusion of a part of an intron into the mRNA, are the most optimal DNA variants for splice modulation ASO therapy. When evaluating genetic databases, these variants are rare. About 9% of all variants reported in the Human Gene Variant Database (HGMD) were reported as splicing variants (18.904/20.9911) [20]. These include all variants of the exonic splice enhancer, donor sites, acceptor sites, and deep intronic splice sites. A query of the Leiden Open Variation Database (LOVD) on March 5, 2021 searching for all pathogenic and likely pathogenic variants that are located in an intron >40 nucleotides remote from the exon/intron boundary resulted in 155 unique variants. This represents 0.13% of the total number of pathogenic (97.142) and likely pathogenic variants (25.289) reported in LOVD.

However, it is highly likely that cryptic splicing variants are under-reported [21]. The main reason for this is that they are often not looked for by standard genetic diagnostic analyses—in particular as the intronic space is not systematically enriched and analyzed by non-WGS diagnostics, which is still standard in many routine diagnostic centers. At the same time, cryptic splicing variants have been reported for many different genes [22], suggesting that for most autosomal recessive diseases or haploinsufficiency diseases, cryptic splicing can be the pathogenic cause. For some diseases, cryptic splicing variants are in fact very common such as *POLR3A* variants for recessive spastic ataxia [23], *ATM* variants for Ataxia telangiectasia [24], *ABCA4* for Stargardt diseases [25], and *NF1* variants for neurofibromatosis [26].

Although searching for intronic variants resulting in cryptic splicing events is challenging, we propose to prioritize patients with recessive diseases for whom only a pathogenic variant has been found on one allele for further analysis. This approach might have a high yield. For example, a pathogenic deep intronic variant was found in 3/6 families with Leber Congenital Amaurosis for whom one pathogenic *RPGRIP1* variant had already been detected [27]. A deep intronic pathogenic variant was also discovered for a family with autosomal recessive polycystic kidney disease where one pathogenic variant in the *PKHD1* gene was found already [28]; or for families with severe multisystemic optic atrophy syndromes where the one pathogenic variant in the *OPA1* gene found already did not explain the severe multisystemic disease course [14,29].

Alternatively, patients whose phenotype strongly suggests a pathogenic variant in a specific gene and whose standard diagnostic workup did not reveal a causal variant could also be studied. This was recently successfully done to identify deep intronic *BRCA1* and *BRCA2* variants in families with early onset breast cancer [30], deep intronic *SMCHD1* variants in families with Facioscapulohumeral muscular dystrophy type 2 (FSHD2) [31], or a deep intronic *WDR45* variant in a family with beta-propeller protein-associated neurodegeneration (BPAN) (Roeben *et al.*, in preparation).

## Regulatory and Legal Perspectives: Guidance for a Promising n-of-1 Approach in Europe

### Regulatory background

In the European Union, the Orphan Regulation ((EC) 141/2000) incentivizes development and marketing of designated orphan medicines, for example, by granting “market exclusivity” for a period of 10 years. Furthermore, orphan medicines are explicitly included in the general legal framework for authorization and supervision of medicines for human and veterinary use (regulation (EC) 726/2004). Marketing authorization under “exceptional circumstances” (article 14(8)) can be granted when collection of comprehensive data on the efficacy and safety under normal conditions of use is impossible, for example, because the intended indication is extremely rare or collection of such data would be unethical.

Outside of centralized marketing authorization, compassionate use programs (article 83 of (EC) 726/2004) enable patients with a chronically or seriously debilitating or life-threatening disease to access unlicensed medicinal products if these are either subject of a marketing authorization application or undergoing clinical trials. In addition, named patient use allows provision of unlicensed medicinal products to single patients to “fulfill special needs” (article 5 (1) of (EC) 2001/83) under direct responsibility of an authorized health care professional on the basis of purely therapeutic considerations. It is important to note that while the European Union grants these two exceptions from centralized marketing authorization, it is at the discretion of the Member States to create the legal framework for implementation. Thus, national regulations concerning manufacturing and application of medicines under compassionate use and named patient use programs vary across European Member States.

For Advanced Therapy Medicinal Products (ATMPs), the so-called ATMP regulation ((EC) 1394/2007) provides regulation for manufacturing and application of ATMPs both within and outside of centralized market authorization procedures. ATMPs encompass gene therapy medicinal products, somatic cell therapy medicinal products, tissue-engineered products, and combined products (tissue or cells associated with a device). The assignment of ultimate regulatory responsibility for ATMPs follows the same principle as laid out above. For ATMPs that are outside of centralized market authorization, Member States have to provide the respective legal provisions that regulate manufacturing and use of these ATMPs, and their manufacturing and use are thus restricted to the respective single Member State. Indeed, the “hospital exemption,” defined in article 28(2) of (EC) 1394/2007, allows manufacturing and application of ATMPs, which are not routinely manufactured to specific quality standards and are used in a hospital in the same Member State under the sole professional responsibility of a medical practitioner on an individual medical prescription of a medicinal product prepared specifically for an individual patient. The manufacturing of these medicinal products is then under the authorization by the competent authority of the EU Member State. For example, in Germany the hospital exemption was implemented in the German Medicines Act (AMG) as Section 4b “Special provisions governing advanced therapy medicinal products” [32].

**FIG. 2.**
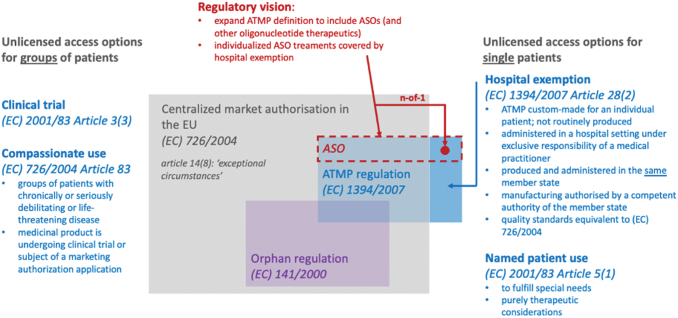
Regulatory framework for access to authorized and unlicensed medicinal products. Color images are available online.

ASOs, however, are chemically synthesized, and as such do not fall under the definition of a Gene Therapy Medicinal Product (GMTP) or ATMP as the definition of a GMTP pertains exclusively to biological medicinal products (part IV of Annex I to (EC) 2001/83) (Fig. 2).

### Regulatory options for n-of-1 ASOs

Individualized ASOs are intended for application in a single patient only. As such, even the concept of market authorization under exceptional circumstances seems not feasible as it involves extra effort and expenses, while definite evidence can still not be generated in single-patient applications. This currently leaves only named patient use of these experimental medicines. While production of ASOs as “investigational medicinal products for human use” is regulated by Commission Delegated Regulation (EU) 2017/1569, most of the other aspects of regulatory oversight over named patient use depend on national law. In Germany, indication of named patient use is justified in the German criminal law code (“Strafgesetzbuch,” StGB) as “justified emergency” (§ 34 StGB). However, clear regulatory pathways governing requirements for the treatment rationale and preclinical evidence, standards for toxicological evaluation and safety aspects, and ethical considerations are missing. In the Netherlands, the named patient treatment application (artsenverklaring) needs to be filed with the inspection for health care (Inspectie Gezondheidszorg en Jeugd). We expect that the regulations vary for other European Member States [33], and that guidelines are lacking for the majority if not all.

### ATMP hospital exemption as a blueprint for an ASO regulation

This regulatory gap is in stark contrast to the clear path laid out for ATMPs by the ATMP regulation ((EC) 1394/2007). Here, the hospital exemption not only allows application of ATMPs outside of centralized marketing authorization—a necessary exception for ultrarare and single-patient diseases—but also specifies that quality standards should be equivalent to regulation (EC) 726/2004. ATMP manufacturing and application even under the hospital exemption is hereby firmly placed under national regulatory oversight. In Germany, this regulation is implemented in the German Medicines Act (AMG), and the Paul Ehrlich Institute is defined as the competent federal authority. In keeping with the European regulation (article 28(2) of (EC) 1394/2007), AMG § 4b issues special provisions for ATMPs that are prescribed by a doctor as an individual preparation for a single patient, are not routinely manufactured in accordance with specific quality standards, and are used in a specialized health care facility under the professional responsibility of a physician. Interestingly, the AMG here specifies that this applies to medicines that are manufactured and used on such a small scale that it is not to be expected that sufficient clinical experience can be gained to enable the medicinal product to be comprehensively evaluated, or which have not yet been manufactured and used in sufficient numbers to allow their comprehensive evaluation. To obtain authorization for application of ATMPs, details of the specialized health care facilities in which the medicinal product is to be used, the number of planned applications or patients per year, details of the dosage, and details of the risk management plan need to be specified. Although the ATMP regulation thus appears to perfectly fit the regulatory requirements for n-of-1 ASO therapies, failure of ASOs to meet the definition of an ATMP places them outside of the pertinent European and national regulation under the poorly defined umbrella of named patient use.

In light of the potentially far-reaching consequences of genetic therapies applying ASOs, their therapeutic potential and thus the goal of making them more widely available through a platform for individualized genetic therapies, a regulatory gap is evident at both European and national (German) levels in several areas:

- Regulatory framework for manufacturing and application of ASOs outside of centralized marketing authorization procedures, equivalent to the hospital exemption as defined for ATMPs (article 28(2) of (EC) 1394/2007).
- Uniform standards to assess quality, preclinical and clinical aspects of ASOs, similar to the existing guideline for GTMPs (EMA/CAT/80183/2014, current effective version 22 March 2018) risk–benefit assessment.
- Clear regulations on liability for the use of experimental ASO therapies outside of centralized marketing authorization procedures; under the named patient program, liability is currently attributed solely to the treating physician despite the lack of regulations governing the use of individualized ASO therapies.
- Framework for reimbursement of individualized therapies by the national health care systems; in most European countries reimbursement is coupled to market authorization, and reimbursement for unlicensed therapies is therefore only granted on a case-by-case basis.

## Ethical Considerations: Systematic Guidance for Developing n-of-1 Treatment Strategies in Europe

### n-of-1 treatments: systematic strategies between patient care and clinical research

The gold standard of clinical research—large randomized controlled trials (RCTs)—cannot be applied to evaluate ASO treatments for single or very few patients with rare diseases. Instead, such highly individualized n-of-1 treatments could be directly applied in the clinical setting as an informal “trial of therapy” [34] (German: *individueller Heilversuch*, Dutch: *artsenverklaring*), which can ethically be justified if no proven therapies for the patient's condition exist (cf. Declaration of Helsinki #37 [35]). However, such clinical treatment attempts are guided by the goal to optimize individual patient care, and are not *per se* geared to gain generalizable knowledge about the new treatment. There has thus been increasing interest over the last few years in more formalized approaches that go beyond these informal “trials of therapy,” which allow a more thorough and reliable evaluation of highly individualized therapies through n-of-1 trials [34,36,37].

Typical designs of n-of-1-trials involve single subjects periodically receiving active treatments or placebo in an ABAB multiple crossover design, ideally in a randomized and blinded manner. This standard n-of-1 trial design, however, is not applicable to disease-modifying therapies such as ASO treatments, as they aim to alter the course of the disease with sustained long-term effects, which often persist after stopping the treatment. Thus, novel designs are needed to evaluate the disease-modifying effects of n-of-1 ASO treatments. Such designs may comprise AB *pre-/post* comparisons, with a pretreatment run-in natural history phase ( = phase A), which allows modeling the individualized natural disease trajectory on a single-subject level, followed by the treatment phase ( = phase B). Here, the modeling of actually observed natural history disease trajectory is used to predict a natural history trajectory for each individual subject, and the trajectory under treatment is then compared with this modeled trajectory. This allows the detection of possible treatment-induced modifications of the disease trajectory even on a single-subject level. At the same time, this intraindividual comparative approach allows one to assess potential individual side effects and burdens of the treatment.

The typical purpose of a n-of-1 trial is to determine whether a treatment works in a single subject. However, n-of-1 trials can be considered as “controlled mini trials” (whether AB- or ABAB-controlled or even as a mini-RCT [38]), with the subject providing multiple datasets to an analysis of the control ( = natural history) versus intervention ( = treatment) period on a group level. Thus, several subjects undergoing a similarly designed n-of-1 trial can contribute many datasets to an “aggregated n-of-1 trial”, which might even scale up to the point where the power of the trial can equate to a normal phase 1 trial, just with fewer participants [38]. Such an aggregated n-of-1 trial could be run either *post hoc* by a retrospective aggregation and analysis of various single n-of-1 trials; or it could be prospectively designed, for example, as a phase 1 umbrella trial [39].

Both, informal “trials of therapy” and more formalized n-of-1-trials, raise conceptual and ethical questions: How can we gain generalizable knowledge across several individual applications of ASO treatments? Where is the boundary between patient care and research in a series of ASO treatment attempts? In introducing n-of-1 ASO treatments, two fundamental ethical obligations come together: promoting the individual patient's well-being and gaining generalizable knowledge for the benefit of future patients. While these two ethical obligations do not pose an ethical dilemma *per se*, the challenge remains on how these two obligations can be accommodated in an ethically appropriate manner?

In Table 3, we provide a systematic, ethically informed chart of possible strategies for developing highly individualized treatments such as ASOs with their basic conceptual and ethical features. These range from (i) informal “trials of therapy” in clinical practice (with a clear primary goal of optimizing individual patient care, focused on the specific individual) over (ii) n-of-1 trials (where the goal to gain generalizable knowledge already has more weight, going also beyond the very specific individual) to more aggregated and systematic research approaches such as (iii) aggregated individual “trials of therapy” and (iv) aggregated n-of-1 trials. These four strategies are characterized by an increasing gain of reliable knowledge with less risk of bias (cf. goal in Table 3). In contrast, the actual number of subjects (e.g. 1 or 5 subjects) does, for example not present the critical criterion for distinction.

**Table 3.** Clinical Care and Research Strategies for n-of-1 Treatments: A Conceptual Framework

Characteristic	“Trial of therapy”	N-of-1 trial	Aggregated “trials of therapy”	Aggregated n-of-1 trials
**Goal** (primary/secondary)	Optimize individual patient care >> gain generalizable knowledge	Gain generalizable knowledge > optimize individual care	Optimize individual care (treatment) ≈ generalizable knowledge (analysis)	Gain generalizable knowledge >> optimize individual care
**Setting/design**	Clinical care	Prospective experimental trial design	Clinical care; prospective or retrospective observational design	Prospective experimental trial design
**Study protocol**	No	Yes	Yes	Yes
**Main factors determining the treatment population**	Clinical need	Research requirements (inclusion/exclusion criteria) > clinical need	Clinical need (treatment) < = > research requirements (analysis)	Research requirements (inclusion/exclusion criteria) >> clinical need
**Intervention** (dose, duration, frequency, route)	Individualized	Standardized	Individualized	Standardized
**Outcomes and data collection**	Systematic for individual treatment decisions	Standardized for knowledge gain (including individual treatment)	Systematic for individual treatment decisions (treatment) ≈ standardized for knowledge gain across individuals (analysis)	Standardized for knowledge gain across individuals
**Analysis**	Individual level	Individual level	Individual and aggregated level	Individual and aggregated level
**Informed consent**	IC to treatment	IC to treatment and trial participation	IC to treatment and observational study	IC to treatment and trial participation
**Ethical support and oversight**	HEC	HEC > IRB	IRB (analysis) and HEC (treatment)	IRB
**Publish results?**	yes (case reports and/or registry)	yes (case reports and/or registry)	Yes	Yes

This table provides an elaboration and modification of an earlier, yet different version of classifying n-of-1 treatments [36].

HEC, hospital ethics committee; IRB, institutional review board, IC, informed consent.

### The distinction between patient care and clinical research: ethically valid?

Since the seminal Belmont report [40], the main distinction between patient care and clinical research is based on the activity's goal: while patient care aims at providing optimal care to individual patients, clinical research aims at gaining generalizable knowledge with potential benefit for future patients. Requirements for ethical and regulatory oversight heavily rely on this distinction. The overview of goals in Table 3 shows, however, that this distinction may become difficult when developing highly individualized precision medicine treatments such as n-of-1 ASO treatments (as well as other individualized gene therapies, such as AAV-based therapies, or highly individualized immunotherapies or cancer therapies).

In fact, there are good ethical reasons that providing best possible care to individual patients should inherently be *integrated* with various efforts to gain generalizable knowledge by collecting and analyzing data alongside clinical care [41]. This integration of patient care with clinical research not only contributes to the paradigm of a learning health care system [42], but also appears to be the right approach to evaluating highly individualized precision medicine treatments. Accordingly, even individual “trials of therapy,” which still have the primary goal to promote individual well-being by applying an unproven, but promising treatment, should be accompanied by a systematic collection of data on treatment outcomes, primarily to inform the treatment decisions for benefit of the individual patient, but also for the benefit of future patients. Systematically collecting data in individual “trials of therapy” is thus an *ethical* obligation, as laid out in section 37 of the Declaration of Helsinki: “In the treatment of an individual patient, where proven interventions do not exist or other known interventions have been ineffective, the physician, after seeking expert advice, with informed consent from the patient or a legally authorised representative, may use an unproven intervention […]. This intervention should subsequently be made the object of research, designed to evaluate its safety and efficacy. In all cases, new information must be recorded and, where appropriate, made publicly available” [35].

If it is not the distinction between patient care and research, which is normatively critical, we need other criteria to determine whether and how far ethical and regulatory oversight are necessary [43]. For example, ethical oversight could be tailored to the risks and burdens of both activities, the uncertainties in individual benefit and in how much the research activities require a deviation from standard patient-centered care. In addition to protecting patients' interests, ethical oversight should play a more constructive role in shaping clinical practice according to the ethical obligations of good clinical care and gaining generalizable knowledge. As we have indicated in Table 3, hospital ethics committees (HECs) and institutional review boards (IRBs) could share the responsibility in promoting and safeguarding the fact that integrated care and research activities in the development of novel ASO therapies are carried out according to the required ethical standards [42].

### The need for a registry and standardized outcome assessments across n-of-1 ASO treatments

To implement the evaluations of efficacy and safety and to establish public transparency, as demanded by the Declaration of Helsinki, it would be of high value to register n-of-1 treatments—whether individual “trials of therapy” or n-of-1 trials—in a standardized crosscenter registry (Box 1).

**Box 1.** An n-of-1 Antisense Oligonucleotide Registry: Scientific and Ethical Advantages (Adapted and Refined from [44])

• accumulate evidence for potential beneficial effects
• aggregate information on adverse events
• collect long-term data (eg, also pharmacovigilance data after possible publication of the initial results)
• collect data on ineffective “trials of therapy” or failed n-of-1 trials
• reduce publication bias, which is of particular importance in this emerging field where standard clinical trials are not possible (ie, failed n-of-1 treatments would otherwise likely not be published and made public)
• help to inform and prepare other n-of-1 treatments and larger research trials
• create transparency about ongoing n-of-1 activities which would otherwise be scattered in small numbers around the world
• facilitate coordination of research trials and research groups
• attract pharma companies by informing them in an aggregated fashion about promising versus ineffective disease targets, outcome measures capturing efficacy, etc.

In addition, and complementing the registry, the systematically collected single-subject data should ideally be fed into an aggregated analysis. This research strategy, which we have called “aggregated informal ‘trials of therapy’” (Table 3), could be the conceptually and ethically most appropriate way to introduce and evaluate novel ASO therapies. A first application of an ASO treatment would be guided by medical need and the goal to provide optimal care for the patient ( = informal “trial of therapy”). However, this individualized treatment should then be subjected to a systematic, protocol-based data collection, which can then allow a systematic analysis of an aggregated series of informal “trials of therapy.” Such an aggregated n-of-1 analysis allows one to collectively evaluate ASO treatments of the same target mutation, but—if designed well—also across different target variants (eg, across different splice variants from the same or different diseases). The focus of such an aggregated data analysis across different variants would be the evaluation not of a specific ASO treatment, but of the *general principle* of the ASO treatment (eg, of ASO splice modulation as a treatment principle in RNDs).

**FIG. 3.**
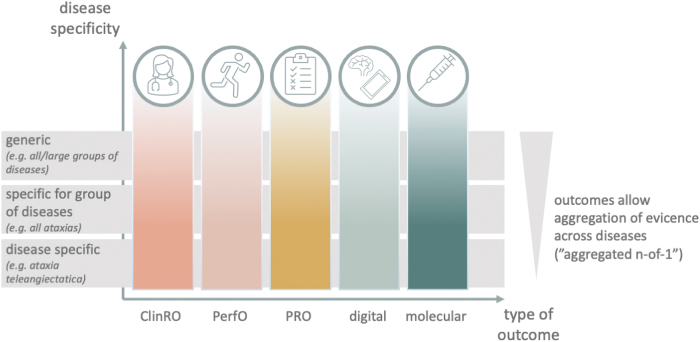
Outcome parameters for individualized therapies. Like in standard clinical trials, outcome evaluation in n-of-1 treatments can include several modalities, including clinician-reported (ClinRO) or patient-reported (PRO) outcomes, performance outcomes (PerfO), digital outcomes (eg, imaging, sensor-based movement or activity analysis, etc.), and molecular outcomes (eg, fluid biomarkers). Each of these outcome modalities may be tailored to a certain level of specificity: disease specific, specific for a group of diseases, or generic. Color images are available online.

These considerations also necessitate rethinking of outcome parameters in n-of-1 treatments. Outcome parameters for individualized therapies can be tailored to different levels of specificity: disease specific, specific for a group of diseases, or generic (Fig. 3). Ideally, outcome measures on all of these levels should be included when monitoring treatments or performing trials in n-of-1 scenarios. This will allow one to maximize the knowledge we can obtain from each of these treatment attempts, whether they are performed in a patient care or clinical research context. When aggregating evidence from multiple trials of treatments or n-of-1 trials, generic outcomes are of particular importance as they can easily be combined across diseases.

## Conclusions

With three unique patients treated with an experimental highly individualized ASO in the United States, this promising approach of n-of-1 ASO treatment now warrants an expansion and scaling up—not only in the United States but in particular also in other continents such as Europe. In summary, the following considerations might help take first steps on this development path in the genetic, regulatory, and ethical domain.

- Splice modulation is a promising cross-disease target mechanism for ASOs. It is amenable to a large number of RNDs, allowing to target in particular deep intronic, but also exonic cryptic splice variants in a wide range of RNDs.
- A regulatory gap, touching on several areas, exists in Europe that places n-of-1 ASO treatments mostly under responsibility of the treating physician (named patient use).
- The ATMP hospital exemption might serve as a blueprint for future ASO regulation; this would require ASOs (and potentially other oligonucleotide therapies) to be included in the definition of an ATMP.
- N-of-1 treatments can be classified as follows: (i) individual trials of therapy, (ii) n-of-1 trials, (iii) aggregated individual trials of therapy, and (iv) aggregated n-of-1 trials.
- For disease-modifying treatments (eg, ASOs), the classical concept of n-of-1 trials—which is still strongly linked to only crossover trial designs—needs to be extended by including novel, innovative trial designs, for example, a *pre-/post* design, with a pretreatment natural history phase.
- Highly individualized treatments such as n-of-1 ASO emphasize the need to integrate patient care with clinical research: each single trial of therapy should inherently be driven to generate generalizable knowledge. Ethical oversight could here be tailored to the risks and burdens of both activities, the uncertainties in individual benefit and in how much the research activities require a deviation from standard patient care.
- There are strong ethical and scientific arguments to (i) establish a global n-of-1 ASO treatment registry and (ii) use outcome measures across different levels of specificity in each n-of-1 treatment—whether it is an informal individual trial of therapy or n-of-1 trial.
